# Tomato fever and COVID 19, a double hit in the Indian health system

**DOI:** 10.1002/iid3.677

**Published:** 2022-07-12

**Authors:** Dattatreya Mukherjee, F. N. U. Ruchika, Nishan B. Pokhrel, Vikash Jaiswal

**Affiliations:** ^1^ Department of Medicine Jinan University Guangzhou China; ^2^ Department of Medicine JJM Medical College Davangere Karnataka India; ^3^ Department of Medicine Tribhuvan University Institute of Medicine Kathmandu Nepal; ^4^ Department of Medicine AMA School of Medicine Makati Philippines

Dear Editor

Two states in India, Kerala and Tamil Nadu, have been battling a new viral flu called tomato fever for the past 2 weeks.^1^ Although only a small part of Kollam has this strain of virus, officials warn of the rampant spread of the disease. Eighty‐two cases of the flu have so far been recorded.^2^ Historically, Tomato fever cases were reported in the past in 2007 in Kerala. Many people were infected at the time in areas of Mudakayam, Varzur, and Kanirapally in Kottayam and Pathinamtita districts, which were earlier infected by Chikungunya.^3^ The alarming facet of this flu is its prevalence in children below the age of 5 years. The term “tomato flu” or “tomato fever” has been given to the disease because of the red‐colored bullous blisters, shaped like tomatoes, that crop up on the affected individuals' bodies.^4^ It is still undetermined whether tomato fever is a viral fever or an aftereffect of Chikungunya or Dengue fever.^4^ The flu is characterized by symptoms like fever, fatigue, red blisters on the skin, rashes, irritation, and dehydration.^4^ The virus may persist in their system for many weeks, even after signs and symptoms of the disease have subsided. Kerala has reported over 58 deaths and hospitalizations due to food poisoning, which has been retrospectively identified as tomato flu.^5^ Another State, Odisha, in the western part of India, has now reported that 26 Children also got affected by tomato flu.^6^ However, some newspapers and experts have suggested the cause of this outbreak to be head foot mouth disease (HFMD) rather than tomato fever.^7^, ^8^, ^9^ Mostly it is caused by Coxsackie Virus A16.^10^ Famous Virologist Dr. Jacob John said that HFMD is caused by two different viruses, Coxsackie A16 and Enterovirus 71.^9^ He also told that the first one is milder and takes a long time to spread.^9^ The common symptoms of HFMD are skin rash on the palm of hands and soles of the foot, dehydration due to mouth sores, and fever.^10^ Due to the absence of substantial scientific literature, the exact cause of the outbreak is still under the microscope.

Even as we are waiting for the end of the coronavirus disease 2019 (COVID‐19) pandemic, this new infection has made its way to Kerala, Tamil Nadu, and Odisha. In various states of India, COVID 19 cases are increasing again. This comes at a time when globally, there is growing concern about a monkeypox epidemic, a viral illness spread by intimate contact with sick people.^11^ Kerala health officials have warned that this disease is contagious and could also spread to other regions. The Kerala Health department is observing the situation closely and taking preventive steps. Anganwadi centers in the affected districts have been closed due to the high number of patients. In the meanwhile, authorities have started village‐wide awareness programs.^12^ Tamil Nadu has established strict rules and regulations to screen travelers from Kerala. To curb the spread, a team of revenue, health, and police officials have been deployed at the Walayar check post located on the Tamil Nadu‐Kerala border to screen people coming from the neighboring state. Karnataka, a neighboring state, has also amped up its surveillance of travelers from the affected regions.

**Figure 1:**
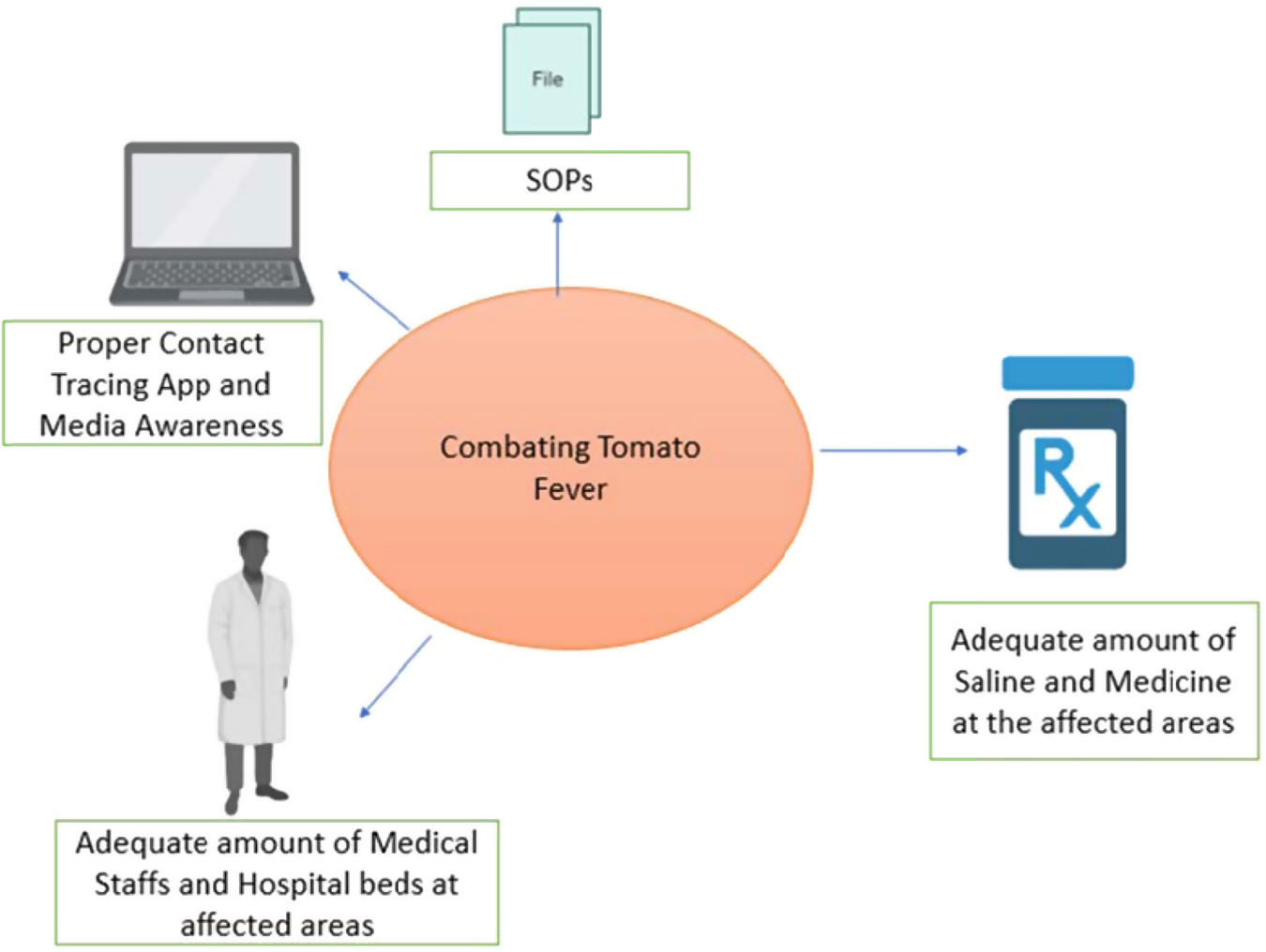
The essential recommendations to combat tomato fever in the early stage. SOPs, standard operating procedures

India is a highly populous country, so early prevention is essential. Strict measures should be taken. The pathogenesis of tomato fever and HFMD^13^ is still unclear. Mass tests are needed in the affected areas. If HFMD is implicated as an etiology, then the child should be quarantined and with proper treatment, the child will recover in 8–10 days.^9^ Health officials warn against at‐home treatment and urge parents to bring their patients to the hospital. Like in all other outbreaks, it is essential to increase the hospital bed numbers in the affected areas along with doctors and medical staff. Regular health camps should be held to screen the children. The state government can create a contact tracing app. According to the website of India's leading health care organizations Narayana Health and Apollo Hospitals, the blister should not be scratched.^14^, ^15^ To prevent the virus from spreading, sanitize sick people's utensils, clothing, and other belongings. To prevent the flu from spreading to other family members, someone infected with it should be kept in isolation. Again as per Narayana Health and Apollo Hospitals, children must be bathed with warm water, and they must be provided water, milk, and juice to drink.^14^, ^15^ Fluid intake serves as a mainstay of treatment to prevent dehydration. All affected rural places in India should get proper medicines and fluids in adequate amounts. Adequate rest is essential throughout the healing period. Those who know someone suffering from the disease around them must keep a safe distance from the patient. Like COVID 19 protocols, proper isolation protocols should also be followed for this disease. The preparations for resisting the flu range from acknowledging its true potential and educating the public to gathering the resources and might of local governments to protect the health and safety of their population. Identification of all affected individuals and areas throughout the country is essential to gauge the extent of the disease spread. Local hospitals, community centers, and accredited social health activists (ASHA) are the main pillars in identifying and reporting the cases. Public awareness of the situation is essential to the battle against this disease outbreak. Using flyers, door‐to‐door education, small village gatherings, and media can help spread awareness by teaching people how to detect symptoms of the flu and its appropriate management. News and social media can be used to spread awarenesses. Striking a balance between disseminating relevant information and panic is vital. Proper traveling guidelines should be followed. The world is already unstable with Monkeypox and COVID 19, and now tomato fever is a new threat. Hence, devising strategic systems early on to deal with flu outbreaks decides the severity of the devastation. All the non‐affected states should be prepared with their standard operating procedures (SOPs) (Figure 1). Public health officials should be priorly prepared. Currently, it's vital to identify the exact etiology of this outbreak. It is high time to know the relation between HFMD and tomato fever. If HFMD is implicated as the etiology, timely interventions are essential, as according to CDC, some patients with this disease develop meningitis.^16^ The prevention, as mentioned above, methods are almost the same for both diseases. As COVID 19 is still going on, any new outbreak indeed affects the mental health of the communities, so proper awareness and community‐based counseling are needed. Furthermore, controlling the outbreaks sooner will surely help the country's economy. A collaboration between the government officials, medical staff, and community is critical in competently addressing the “Tomato flu” outbreak during the COVID 19 time.
